# Non-invasive neurosensory testing used to diagnose and confirm successful surgical management of lower extremity deep distal posterior compartment syndrome

**DOI:** 10.1186/1749-7221-4-4

**Published:** 2009-05-16

**Authors:** Eric H Williams, Don E Detmer, Gregory P Guyton, A Lee Dellon

**Affiliations:** 1 Division of Plastic and Reconstructive Surgery, Johns Hopkins University School of Medicine, Baltimore Maryland, USA; 2 Dellon Institutes for Nerve Surgery, Johns Hopkins University, 3333 North Calvert St. Suite 370, Baltimore, Maryland, 21218, USA; 3 Department of Public Health Sciences, Health System, University of Virginia, Charlottesville, Virginia USA; 4 Greater Chesapeake Orthopedic Surgery, 3333 North Calvert St, 4th Floor, Baltimore, Maryland, 21218, USA

## Abstract

**Background:**

Chronic exertional compartment syndrome (CECS) is characterized by elevated pressures within a closed space of an extremity muscular compartment, causing pain and/or disability by impairing the neuromuscular function of the involved compartment. The diagnosis of CECS is primarily made on careful history and physical exam. The gold standard test to confirm the diagnosis of CECS is invasive intra-compartmental pressure measurements. Sensory nerve function is often diminished during symptomatic periods of CECS. Sensory nerve function can be documented with the use of non-painful, non-invasive neurosensory testing.

**Methods:**

Non-painful neurosensory testing of the myelinated large sensory nerve fibers of the lower extremity were obtained with the Pressure Specified Sensory Device™ in a 25 year old male with history and invasive compartment pressures consistent with CECS both before and after running on a tread mill. After the patient's first operation to release the deep distal posterior compartment, the patient failed to improve. Repeat sensory testing revealed continued change in his function with exercise. He was returned to the operating room where a repeat procedure revealed that the deep posterior compartment was not completely released due to an unusual anatomic variant, and therefore complete release was accomplished.

**Results:**

The patient's symptoms numbness in the plantar foot and pain in the distal calf improved after this procedure and his repeat sensory testing performed before and after running on the treadmill documented this improvement.

**Conclusion:**

This case report illustrates the principal that non-invasive neurosensory testing can detect reversible changes in sensory nerve function after a provocative test and may be a helpful non-invasive technique to managing difficult cases of persistent lower extremity symptoms after failed decompressive fasciotomies for CECS. It can easily be performed before and after exercise and be repeated at multiple intervals without patient dissatisfaction. It is especially helpful when other traditional testing has failed.

## Introduction

Chronic exertional compartment syndrome (CECS) is defined as a condition in which exercise or heavy exertion creates elevated pressures within the closed space of an extremity muscular compartment which subsequently causes consistently recurring symptoms and/or disability by progressive impairment of the neuromuscular function of the involved compartment [1-6]. The diagnosis of CECS is primarily made on careful history that demonstrates consistent appearance of symptoms in the same compartments in the lower extremities with exertion. Symptoms may consist of an aching pain, squeezing sensation, sharp pains, or possible paresthesias in the feet. It is not uncommon for bilateral mirror image compartments to be involved. Confirmation of the diagnosis is generally made with direct invasive intra-compartmental pressure measurements [1,4,7,8]. We present a case where non-invasive, non-painful neurosensory testing successfully diagnosed the problem of exertional compartment syndrome and was used to help guide and document successful management of the disorder in a patient with suspected deep distal posterior compartment syndrome.

## Case report

A 25 year old male was originally seen in our office after the diagnosis of chronic exertional compartment syndrome (CECS) of the anterior and lateral compartments had been made by invasive pressure measurements of those compartments. He was originally referred to our office for the treatment of chronic leg pain due to a neuroma of a superficial peroneal nerve, injured during an anterior and lateral compartment fasciotomy to treat his CECS. This painful neuroma was treated successfully by neuroma resection and implantation of the proximal end of the superficial peroneal nerve into the extensor digitorum communis muscle [9]. His anterior and lateral compartment pain had resolved with the original fasciotomies. He was then discharged from our care.

He returned to our office one year later with complaints of bilateral exercise induced pain in the backs of his legs from the lower calf to the ankle that he stated felt "just like the front of my legs did, though slightly less intense." After five minutes of running he began to complain of tightness and a dull aching pain that progressed to severe pain eventually causing him to stop exercising. His pain was also associated with paresthesias and numbness in the soles of his feet. The pain and numbness persisted for five to ten minutes after stopping his exercise, but the tightness lasted longer.

On exam, the patient was an athletic appearing male with normal pulses in dorsalis pedis and posterior tibial vessels. He was tender to pressure applied immediately posterior to the tibia overlying the distal deep posterior compartment. He had no tenderness to percussion of the tibia itself or to palpation of the tibial edge. He was not tender in the midline of the posterior calf over the proximal tibial nerve [10]. His gastrocnemius muscle was slightly tender. He did have a Tinel sign over both tarsal tunnels with radiation to the sole of his feet.

**Figure 1:**
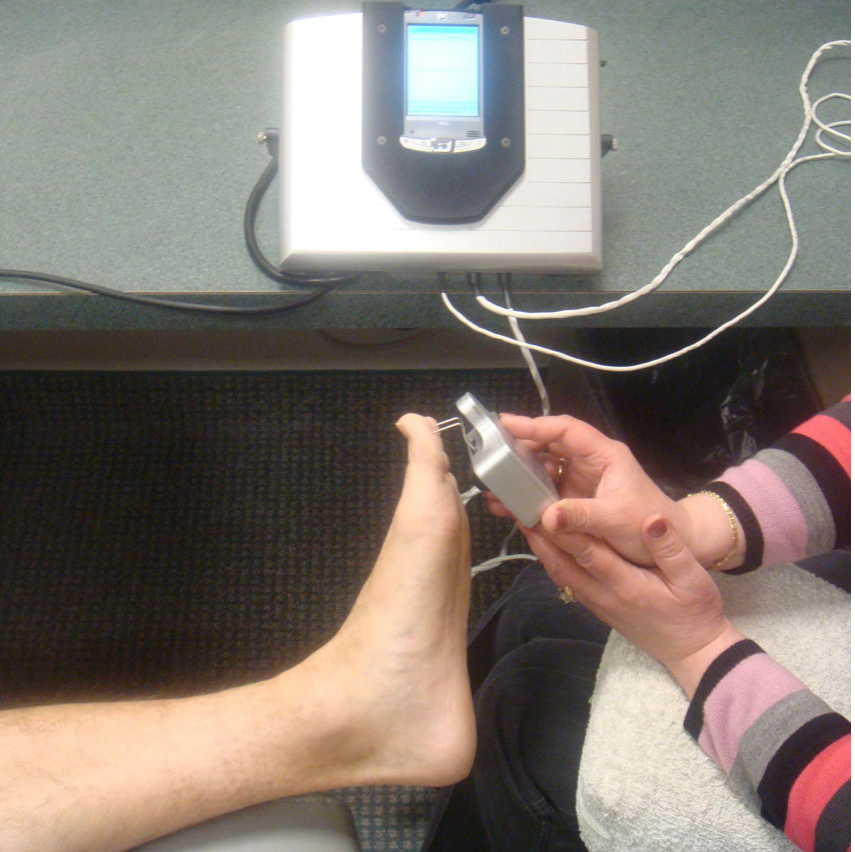
**Measurement of 2 point discrimination in great toe which is in the distribution of the medial plantar nerve branch of the tibial nerve with the use of the Pressure Specified Sensory Device™ (Sensory Management Services, LLC, Baltimore, Maryland)**. This obtains a true measurement of the distance that a patient can feel two distinct points and the pressure which is required to feel those two points.

Due to his symptoms of exercise induced numbness and paraesthesias, non-invasive, non-painful neurosensory testing was performed with the Pressure Specified Sensory Device™ (Sensory Management Services, LLC, Baltimore, Maryland, USA) at rest to measure base line cutaneous pressure thresholds for one and two point static touch and to measure two point discrimination in the skin innervated by medial plantar and medial calcaneal branches of the tibial nerve (Figures 1. and 2). The anterior lateral dorsum of the foot and the dorsal web-space between the first and second toe – the usual distribution of the superficial peroneal and deep peroneal nerve branches respectively – were also measured. The study was repeated immediately after 10 minutes of running on a treadmill – the time interval to reproduce his symptoms. Following the running, there was widening of two point discrimination in the distribution of the calcaneal nerve and the medial plantar nerve indicating loss of large fiber tibial nerve function suggesting the diagnosis of exertional compartment syndrome of the deep posterior compartment causing compression of the tibial nerve (Table 1).

**Table 1:** Neurosensory Measurements Before & After Stress Testing

Cutaneous Pressure Thresholds for Static Two-Point Discrimination*						
	Prior to 1^st ^Posterior Distal Compartment Release *(A)*		After 1^st ^Posterior Distal Compartment Release *(B)*		After 2^nd ^Posterior Distal Compartment Release *(C)*	

RIGHT LEG						
						
Tibial Nerve	Before Exercise	After Exercise	Before Exercise	After Exercise	Before Exercise	After Exercise

Hallux Pulp						
mm	10	15	8	12	5	5
*gm/mm*^2^	*43*	*60*	*46*	*52*	*63*	*66*
Medial Heel						
mm	11	15	8	12	8	5
*gm/mm*^2^	*40*	*58*	*82*	*79*	*96*	*68*
Peroneal Nerve						

1^st ^web space						
mm	5	5	8	10	5	5
*gm/mm*^2^	*60*	*88*	*60*	*68*	*53*	*56*
Dorsolateral**	NA	NA	NA	NA	NA	NA
						
LEFT LEG						
						
Tibial Nerve						

Hallux Pulp						
mm	4	8	10	10	5	5
*gm/mm*^2^	*38*	*97*	*40*	*52*	*79*	*75*
Medial Heel						
mm	5	15	8	8	5	5
*gm/mm*^2^	*45*	*82*	*92*	*69*	*52*	*64*
Peroneal Nerve						

1^st ^web space						
mm	5	5	8	8	5	5
*gm/mm*^2^	*35*	*77*	*95*	*80*	*73*	*52*
Dorsolateral						
mm	7	7	7	7	7	7
*gm/mm*^2^	*37*	*78*	*90*	*79*	*53*	*71*

*Two-point static-touch; normative values in the foot for someone less than 45 years of age have a pressure of about 15 gm/mm^2 ^to discriminate one from two static points at 6 mm distance apart. ** The right superficial peroneal nerve was resected previously and the anterior and lateral compartments released previously.

A) Interpretation: the distance required to discriminate one from two point static-touchincreased for the tibial nerve on both the right and left sides after exercise, consistent with bilateral (right worse than left) posterior compartment syndrome. Note that the peroneal nerve measurements on the left and right did not change, and that the anterior and lateral compartments had been released previously.

B) Interpretation: There is still an increase in the right tibial nerve measurements for discrimination of one from two point static-touch, indicating that despite fasciotomy of the deep compartment on the right, there is still compression of the tibial nerve in the distal deep compartment. Neurosensory testing demonstrates that another fasciotomy is still required. The lack of change in left tibial nerve may be a timing phenomenon as the right leg was tested first after the patient stopped running.

C) Interpretation: After complete decompression of the deep distal posterior compartment bilaterally, there is now no increase in the distance required to discriminate one from two static-touch points, consistent with complete release of the deep distal posterior compartments and return of normal tibial nerve function.

**Figure 2:**
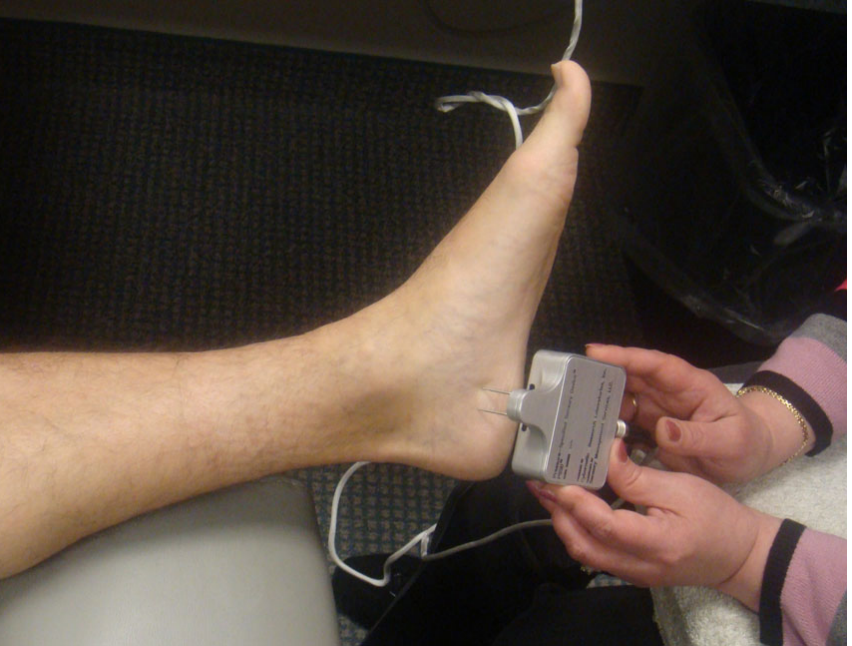
**Measurement of 2 point discrimination in the medial heel which is in the distribution of the medial calcaneal nerve branch of the tibial nerve with the use of the Pressure Specified Sensory Device™ (Sensory Management Services, LLC, Baltimore, Maryland)**. This obtains a true measurement of the distance that a patient can feel two distinct points and the pressure which is required to feel those two points.

To confirm the diagnosis, traditional invasive, immediate, post-exercise compartment pressures of the superficial and deep posterior compartments were obtained using a device with a side port needle measurement system (Stryker Instruments, Kalamazoo, Mich.). The superficial posterior compartment (SPC) measured 40 mmHg on the right and 24 mmHg on the left. The deep posterior compartment (DPC) measured 62 mmHg on the right and 28 mmHg on the left. To rule out other causes of posterior leg pain an MRI was performed and demonstrated no vascular anomalies, no evidence of stress fractures, medial tibial periostitis, tumors, or other abnormalities.

Bilateral superficial posterior and deep distal posterior fasciotomies were performed through a proximal and distal two incision medial approach. Postoperatively, the patient recovered without incident. However, at three months he still complained of similar symptoms, but they were more isolated to the posterior distal half of the lower extremity over the distal deep compartment muscles. The patient's exam still demonstrated pain with compression just posterior to the tibia in the lower half of his legs. Due to his complaints of persistent pain and numbness, his non-invasive neurosensory testing was repeated before and after running 10 minutes on a treadmill (Table 1). Again he demonstrated loss of two point discrimination in the calcaneal and medial plantar nerve that suggested continued tibial nerve dysfunction brought on by exertion.

Therefore he was taken back to the operating room for a repeat fasciotomy of the distal deep compartments. It was discovered that the patient had an unusual anatomic variant of his deep distal compartment as described by Detmer [11], and therefore the compartment had not been fully released during the first operation. The soleus muscle wrapped around medial side of the tibia unusually far, and it completely obscured the deep distal compartment. The fascia that had originally been released turned out to be the fascia overlying the unusually large and medially placed soleus. Only after peeling the soleus completely off the medial edge of the tibia in the distal lower leg was a second deeper layer of thickened fascia found beneath it. This too was released longitudinally to open the true deep distal compartment that encased the posterior tibial neurovascular bundle, the flexor digitorum longus, posterior tibialis, and flexor hallucis muscles.

The patient recovered well from his second operation and was allowed to progress in his exercise regimen starting three weeks after surgery. After his first attempted posterior distal compartment release, he was able to run only a half of a mile before he would need to rest and allow his legs to recover. Three months after his second posterior distal compartment release, he was able to run over three miles with out resting. At 15 months after the second posterior distal compartment fasciotomy, the patient states that he had a 90% improvement in the numbness and posterior leg pain since surgery.

We tested him a third time with the non-invasive neurosensory testing before and after running on a treadmill for 12 minutes and this demonstrated minimal change in two point discrimination indicating minimal change in tibial nerve function, thus demonstrating resolution of nerve compressions caused by his deep distal posterior exertional compartment syndrome.

## Discussion

To our knowledge this is the first case where non-invasive neurosensory testing with the Pressure-Specified Sensory Device™ was used during a provocative test to assist in making the diagnosis and then to help guide surgical management of CECS in an athlete.

The gold standard for diagnosis of CECS is invasive intra-compartmental pressure measurements before, during, and/or after exercise with a wick catheter, slit catheter, or sideport needle [1,4,12]. In addition to elevated pressures seen before, during, and after exercise, there is a delayed return of the intracompartamental pressure to base line when compared to controls [13]. This invasive technique caries with it some discomfort and a small risk of injury to neurovascular structures, furthermore, it may be difficult to tell exactly where the tip of the needle is measuring [1,6,12]. Non-invasive techniques including magnetic resonance imaging, near-infrared spectroscopy, and laser doppler flowmetry, have been described to diagnose CECS in the lower extremities [6,12,14,15]. Several studies have successfully used non-invasive vibration thresholds to diagnose acute compartment syndrome [16,17]. Progressive loss of motor strength was used to demonstrate CESC non-invasively in the upper extremity [18].

Pathophysiologic mechanisms underlying the cause of this syndrome are not fully understood, but generally it is believed that exercise causes an abnormally high intra-compartmental pressure, thus impairing local tissue perfusion and, therefore, causing ischemic pain [5,12,15,19]. However, there is some evidence that ischemia may not be the underlying mechanism of pain [7,14]. Matsen and colleagues studied the effect of compartment pressure on motor nerve conduction velocity, compound muscle-action potential amplitude, sensation to light touch and pin prick [20]. They found a "consistent sequence in the appearance of abnormalities in neuromuscular function during compression." Subjective numbness appeared first followed by hypesthesia to light touch and pinprick, and then motor weakness [20]. This work supports the use of sensibility testing as a means to detect early changes in compartment syndromes.

The function of large myelinated nerve fibers measured by the detection of vibratory sensation has been shown to be a sensitive indicator of acute compartment syndrome as well as chronic nerve compression and nerve regeneration [8,16,17,21,22]. Although vibratory stimulation with a tuning fork or vibrometer is clinically useful, the major drawback is that this form of stimulation sets up a waveform stimulus and will potentially stimulate nerve fibers outside the field of interest and lead to potential misinterpretation [23].

The Pressure-Specified Sensory Device™ offers the clinician, reliable, valid quantitative measurements of pressure threshold and nerve fiber density data by asking the patient to indicate at what distance he can feel two distinct pressure points to the skin. This distance between the points is an indication of the functional nerve fiber density, while the pressure required to feel those two different points is a measure of sensory fiber threshold [23-26]. Neurosensory testing with the Pressure-Specified Sensory Device™ has been proven to be more sensitive and specific than either vibration or Semmes-Weinstein monofilaments in identifying large fiber peripheral nerve dysfunction in patients with chronic nerve compression and peripheral neuropathy [23-25].

The limitations of this technique are that neurosensory testing is a subjective test rather than a purely objective one. It requires a cooperative and truthful patient and a trained technician to perform it. At this time we do not have clinical normative values that describe what amount of sensory change is considered to be pathologic, and further testing needs to be performed.

Neurosensory testing also needs to be performed quickly after the patient stops the exercise in order to pick up the changes in reversible sensory change. It is currently unknown how long these sensory changes can be detected with this device, and clinical study needs to be performed to better determine this. With regards to this particular patient, testing was performed on both feet within 4–5 minutes of stopping his exercise.

Clearly it must be emphasized that this represents only a single case report and further studies to determine population norms, control values, and to determine clinically significant sensory changes must to be performed to prove that this is a useful technique to use for routine purposes to diagnose and follow patients with complaints consistent with CECS.

## Conclusion

With an accurate, valid, non-invasive measurement system, it may be more important to determine treatment based end organ function of the most sensitive organ – the nerve – rather than on pressures in the compartments involved with CECS. If one could accurately determine the real-time function of the peripheral nerve the compartment then one could begin to refine the clinical treatment of patients with suspected CECS.

While compartment pressure measurements are a reliable method of evaluation of patients with suspected CECS, in this report, neurosensory testing demonstrated that a non-painful, non-invasive method was also helpful in directing care in a patient with CECS.

## Abbreviations

CECS: Chronic exertional compartment syndrome.

## Competing interests

One author, ALD has a proprietary interest in the Pressure- Specified Sensory Device ™, and the company Sensory Management Services, LLC that markets it.

## Authors' contributions

EHW: writing, design, interpretation of data, direct patient care, DED: design, patient care, intellectual content, GPG: design, direct patient care, acquisition of data, intellectual content, ALD: writing, interpretation of data, intellectual content

## Consent

Written informed consent was obtained from the patient for publication of this Case report and accompanying images.
